# The incidence, risk factors and prognosis of acute kidney injury in severe and critically ill patients with COVID-19 in mainland China: a retrospective study

**DOI:** 10.1186/s12890-020-01305-5

**Published:** 2020-11-09

**Authors:** Ling Sang, Sibei Chen, Xia Zheng, Weijie Guan, Zhihui Zhang, Wenhua Liang, Ming Zhong, Li Jiang, Chun Pan, Wei Zhang, Jiaan Xia, Nanshan Chen, Wenjuan Wu, Hongkai Wu, Yonghao Xu, Xuesong Liu, Xiaoqing Liu, Jianxing He, Shiyue Li, Dingyu Zhang, Nanshan Zhong, Yimin Li

**Affiliations:** 1 Department of Pulmonary and Critical Care Medicine, State Key Laboratory of Respiratory Diseases, Guangzhou Institute of Respiratory Health, The First Affiliated Hospital of Guangzhou Medical University, Guangzhou, China; 2 Department of Critical Care Medicine, The First Affiliated Hospital of Zhejiang University, Zhejiang, Hangzhou China; 3 Department of Thorax Surgery, State Key Laboratory of Respiratory Diseases, Guangzhou Institute of Respiratory Health, The First Affiliated Hospital of Guangzhou Medical University, Guangzhou, China; 4 Department of Critical Care Medicine, Zhongshan Hospital Fudan University, Shanghai, China; 5 Department of Critical Care Medicine, Xuanwu Hospital, Capital Medical University, Beijing, China; 6 Department of Critical Care Medicine, Zhongda Hospital, Southeast University, Nanjing, China; 7 Emergency Department, the 900th Hospital of Joint Service Corps of Chinese PLA, FuZhou, China; 8 Department of tuberculosis, Wuhan Jinyintan Hospital, Wuhan, China; 9 Department of Respiratory and Critical Care Medicine, Wuhan Jinyintan Hospital, Wuhan, China; 10 Department of Critical Care Medicine, Wuhan Jinyintan Hospital, Wuhan, China; 11 State Key Laboratory of Respiratory Disease, National Clinical Research Center for Respiratory Disease, The First Affiliated Hospital of Guangzhou Medical University, Guangzhou Medical University, Guangzhou, China; 12 Research Center for Translational Medicine, Wuhan Jinyintan Hospital, Wuhan, China; 13 Joint Laboratory of Infectious Diseases and Health, Wuhan Institute of Virology and Wuhan Jinyintan Hospital, Chinese Academy of Sciences, Wuhan, China

**Keywords:** Coronavirus disease 2019, Acute respiratory distres, Syndrome, Acute kidney injury, Intensive care unit, Mechanical ventilation

## Abstract

**Background:**

The clinical correlates, prognosis and determinants of acute kidney injury (AKI) in patients with coronavirus disease 2019 (Covid-19) remain largely unclear.

**Methods:**

We retrospectively reviewed medical records of all adult patients with laboratory-confirmed Covid-19 who were admitted to the intensive care unit (ICU) between January 23rd 2020 and April 6th 2020 at Wuhan JinYinTan Hospital and The First Affiliated Hospital of Guangzhou Medical University.

**Results:**

Among 210 patients, 131 were males (62.4%). The median Age was 64 years (IQR: 56–71). Of 92 (43.8%) patients who developed AKI during hospitalization, 13 (14.1%), 15 (16.3%) and 64 (69.6%) were classified as being at stage 1, 2 and 3, respectively. 54 patients (58.7%) received continuous renal replacement therapy. Age, sepsis, nephrotoxic drug, invasive mechanical ventilation and elevated baseline serum creatinine levels were associated with the occurrence of AKI. Renal recovery during hospitalization was identified among 16 patients with AKI (17.4%), who had a significantly shorter time from admission to AKI diagnosis, lower incidence of right heart failure and higher ratio of partial pressure of oxygen to the fraction of inspired oxygen. Of 210 patients, 93 deceased within 28 days of ICU admission. AKI stage 3, critical disease, greater Age and the lowest ratio of partial pressure of oxygen to the fraction of inspired oxygen being < 150 mmHg were independently associated with death.

**Conclusions:**

Among patients with Covid-19, the incidence of AKI was high. Our findings of the risk factors of the development of AKI and factors associated with renal function recovery may inform clinical management of patients with critical illness of Covid-19.

## Background

Since December 2019, the outbreak of coronavirus disease 2019 (Covid-19) has resulted in over 30.6 million laboratory-confirmed cases and 950,000 death cases worldwide as of September 20th 2020 [1]. The clinical manifestations of Covid-19 have been heterogeneous, ranging from asymptomatic viral carriers to critically ill cases [2, 3]. The clinical outcomes of critically ill patients have been poor, with the mortality rate being 61.5% within 28 days [4]. Identification of the risk factors of death would be crucial to inform clinical decisions to guide early triage of patients for more intensive monitoring. Previous studies have documented that greater age and the presence of comorbidities correlated significantly with death in patients with Covid-19 [5]. Few studies, however, have focused on the systemic complications and the clinical outcomes of Covid-19.

Acute kidney injury (AKI) is a heterogeneous disease and a common complication in critically ill patients. AKI reportedly correlated with poor clinical outcomes in patients with Covid-19 [2–4, 6, 7]. Nonetheless, the clinical correlates, prognosis and determinants of AKI in patients with Covid-19 remain largely unclear. Early identification and intervention would help improve the clinical outcomes of patients with Covid-19 who were prone to develop AKI [8, 9]. Therefore, we performed a retrospective study to evaluate the incidence, risk factors and prognosis of AKI in severe and critically ill patients with Covid-19.

## Methods

### Patients

We reviewed medical records of all adult patients (> 18 years) with laboratory-confirmed Covid-19 who were admitted to the intensive care unit (ICU) between January 23rd 2020 and April 6th 2020 at Wuhan JinYinTan Hospital and The First Affiliated Hospital of Guangzhou Medical University (designated hospitals for admitting patients with Covid-19). The severe acute respiratory syndrome coronavirus-2 (SARS-CoV-2) RNA was detected by using reverse-transcription polymerase chain reaction (RT-PCR) for laboratory diagnosis of Covid-19. The samples consisted of throat swabs, nasal swabs or endotracheal aspirate. All patients met the criteria of having severe or critical Covid-19 according to the Chinese guidelines [10]. The Ethics Committees of both hospitals approved the study protocol. Informed consent was waived due to the nature of the retrospective study.

### Data extraction

The clinical data from 210 cases, including patient demographics, clinical symptoms and signs, laboratory findings, treatment [including respiratory supports, use of medications and continuous renal replacement therapy (CRRT)] and clinical outcomes, were extracted from the electronic records by two independent intensivists (LS and XZ) who subsequently cross-checked for data accuracy. The disagreement was further adjudicated by a third independent reviewer (YML). All data were entered into the computerized database for further statistical analyses.

### Study definitions

Severe Covid-19 met any of the following criteria: (1) Respiratory distress, defined as the respiratory rate ≥ 30 times/min, with cyanos;s; (2) Arterial digital oxygen saturation ≤ 93% (at ;oom air); (3) the ratio of partial pressure of oxygen to the fraction of inspired oxygen (PaO_2_/FiO_2_) ≤300 mmHg (1 mmHg = 0.133 kPa). Critical Covid-19 met any of the following criteria: (1) Respiratory failure requires mechanical ventilation; ;2) Shock; (3) Multiple organ failure requires ICU life support [10]. The diagnosis of shock was made if patients represented with at least two of the following conditions: The systolic blood pressure being 13.3 kPa (100 mmHg);or lower; (2) The pulse pressure being 4.0 kPa (30 mmHg); or lower; (3) Symptoms consistent with the presentation of shock (i.e. poor peripheral circulation and tachycardia); (4) Urine volume being less than 25 ml/h; (5) Blood lactic acid being 3 mmol/L or greater; (6) Cardiac index being 2.5 L/(min.m^2^) or lower.

The definition, severity staging and clinical management of AKI were based on the *Kidney Disease: Improving Global Outcomes* (KDIGO) classification [9]. The clinical management could also be amended as per the clinical needs at the discretion of the attending clinicians [11, 12]. Recovery of AKI was defined based on Acute Disease Quality Initiative (ADQI) 16 Workgroup: (1) rapid sustained reversal: recovery from AKI within 48 h, (2) late sustained reversal: reversal after 48 h and sustained through 28 days after AKI diagnosis or hospital discharge, (3) relapsing AKI with complete recovery, (4) relapsing AKI without complete recovery, and (5) never recovered. The first three categories were classified as complete renal recovery while the last two categories were classified as renal non-recovery [13]. The recovery of AKI was monitored until patient’s discharge from hospital.

### Study outcomes

The study outcomes mainly consisted of the incidence of AKI and the use of CRRT. We also assessed the risk factors for AKI, the outcomes of renal diseases, and the impact of AKI on the clinical outcomes of Covid-19.

### Statistical analysis

Continuous variables were expressed as the median and interquartile ranges (IQRs) and compared with Wilcoxon rank-sum test. Categorical variables were expressed as counts and percentages, and compared using chi-square test or Fisher’s exact test as appropriate. In clinical practice, the elevation in serum creatinine levels preceded the development of AKI. Therefore, the risk factors for AKI were screened with univariate logistic regression model, and the variables with *P* value of 0.10 or less were considered as the potential risk factors and further imported into the multivariate logistic regression analysis. The AKI and survivor risk model was established by calculating the regression coefficient (β), odds ratio (OR) and 95% confidence interval. The significance threshold was set at a 2-sided *P* value of less than 0.05. All statistical analyses were performed using R version 3.5.1 software (the R Foundation, USA) or SPSS version 17.0 (SPSS Inc., USA).

## Results

### Patient characteristics stratified by the severity of Covid-19

We have initially included 262 patients, and excluded 52 patients (20 patients discharged or died within 24 h, and 32 patients had missing data). Finally, we included 210 patients with complete data, of whom 131 were males (62.4%). The median age was 64 years (IQR: 56–71) **(**Table 1**)**. None of the patients had AKI at admission. The patient characteristics as stratified by the disease severity are shown in Table E1. 60 patients were categorized as having severe disease and the remaining 150 patients as having critical disease upon admission to ICU. The gender and the age were comparable. Diabetes was more common in critical cases than in severe cases (24.7% vs 11.7%, *P* = 0.037). There were, however, no significant differences for other comorbidities between severe and critical cases.

**Table 1:** Clinical characteristics of patients with Covid-19 when stratified by the presence of AKI during hospitalization

Characteristic	Total (***N*** = 210)	AKI (***n*** = 92)	No AKI (***n*** = 118)	***P*** value
**Age, median (IQR), yrs**	**64 (56–71)**	**65 (59–73)**	**62 (54–70)**	**0.021**
**Sex**				0.186
Female	79 (37.6)	30 (32.6)	49 (41.5)	–
Male	131 (62.4)	62 (67.4)	69 (58.5)	–
**Severity of Covid-19**				**< 0.001**
Severe	**60 (28.6)**	**2 (2.2)**	**58 (49.2)**	–
Critical	**150 (71.4)**	**90 (97.8)**	**60 (50.9)**	–
**Comorbidities**				
Hypertension	98 (46.7)	45 (48.9)	53 (44.9)	0.565
Diabetes	44 (21.0)	23 (25.0)	21 (17.8)	0.203
Cardiovascular diseases	23 (11.0)	9 (9.8)	14 (11.9)	0.632
Malignancy	**14 (6.7)**	**10 (10.9)**	**4 (3.4)**	**0.031**
Cerebrovascular disease	12 (5.7)	6 (6.52)	6 (5.1)	0.656
Chronic Kidney Disease	10 (4.8)	6 (6.52)	4 (3.4)	0.290
Chronic Obstructive Pulmonary Disease	5 (2.4)	2 (2.2)	3 (2.5)	NA
Connective Tissue Disease	2 (1.0)	2 (2.2)	0 (0.0)	NA
**Complication**				
Sepsis	**112 (53.3)**	**72 (78.3)**	**40 (33.9)**	**< 0.001**
Right heart failure^a^	**44 (21.0)**	**28 (30.4)**	**16 (13.6)**	**0.003**
Disseminated Intravascular Coagulation	**32 (15.2)**	**27 (29.4)**	**5 (4.24)**	**< 0.001**
**Treatment**				
Drug with Nephrotoxicity	**52 (24.8)**	**38 (41.3)**	**14 (11.9)**	**< 0.001**
Respiratory support: COT	**41 (19.5)**	**2 (2.2)**	**39 (33.1)**	**< 0.001**
Respiratory support: HFN	22 (10.5)	6 (6.5)	16 (13.6)	0.099
Respiratory support: NIV	**29 (13.8)**	**4 (4.4)**	**25 (21.2)**	**< 0.001**
Respiratory support: IMV	**118 (56.2)**	**80 (87.0)**	**38 (32.2)**	**< 0.001**
**Laboratory Findings**^b^				
Baseline serum creatinine (μmol/L)	**69.7 (55.9–85.9)**	**75.5 (59.8–102.8)**	**67.1 (54.3–78.4)**	**0.002**
Maximum PaCO_2_ (mmHg)	**52.0 (39.0–73.8)**	**69.5 (46.8–81.3)**	**45.0 (38.3–57.5)**	**< 0.001**
Maximum PaCO_2_ > = 60 mmHg	**82 (39.05)**	**55 (59.78)**	**27 (22.88)**	**< 0.001**
Minimum PaO2/FiO2 ratio (mmHg)	**109.0 (65.0–207.7)**	**73.0 (62.8–138.6)**	**169.0 (74.3–217.5)**	**< 0.001**
Minimum PaO2/FiO2 ratio < 150 mmHg	**116 (55.24)**	**70 (76.09)**	**46 (38.98)**	**< 0.001**
Maximum IL-6 (pg/ml)	**14.3 (9.9–24.9)**	**17.9 (12.6–33.5)**	**12.3 (8.9–19.6)**	**< 0.001**
Maximum serum ferritin (ng/ml)	**1607.8 (786.2–2001.0)**	**2001.0 (1329.6–2001.0)**	**985.4 (581.8–2001.0)**	**< 0.001**

^a^ Right heart failure: defined according to the clinical diagnosis and ultrasonographic manifestations. & Drugs with Nephrotoxicity: mainly included aminoglycosides, glycopeptides and colistin. ^b^, Data were collected before the development of AKI among patients who had developed AKI; The most abnormal data during hospitalization were presented among patients who did not develop AKI. PaCO_2_: arterial partial pressure of carbon dioxide; PaO_2_/FiO_2_ ratio: the ratio between the arterial partial pressure of oxygen and the inspiratory concentration of oxygen; Data in bold indicated the comparisons with statistical significance

Overall, the most common respiratory support for severe cases consisted of conventional oxygen therapy (COT) and high-flow nasal cannula (HFNC), A significantly larger proportion of patients with critical disease were initiated with invasive mechanical ventilation(IMV). There was, however, no significant difference in the use of non-invasive mechanical ventilation (NIV) between severe and critical cases.**(**Table E1**).**

Of the 210 patients, 194 were from Wuhan JinYinTan Hospital, and 16 patients were from The First Affiliated Hospital of Guangzhou Medical University. Right heart failure, hypoxemia, hypercapnia and higher levels of interleukin-6 were more common in patients from Wuhan JinYinTan Hospital (both *P* < 0.05).**(**Table E2**).**

### The incidence of AKI and use of CRRT, and the risk factors associated with AKI

Of 92 (43.8%) patients who developed AKI during hospitalization, 13 (14.1%), 15 (16.3%) and 64 (69.6%) patients were classified as having stage 1, 2 and 3 disease, respectively **(**Tables 1, 3**)**. 52 patients (56.5%) received CRRT.

Patients with AKI were significantly more likely to be critical cases (97.8% vs. 50.9%, *P* < 0.001), have malignancy (10.9% vs. 3.4%, *P* = 0.031), and develop sepsis (78.3% vs. 33.9%, *P* < 0.001), right heart failure (30.4% vs. 13.6%, *P* = 0.003) and disseminated intravascular coagulation (29.4% vs. 4.2%, *P* < 0.001). Significantly more critical cases than severe cases who received nephrotoxic drugs developed AKI (41.3% vs. 11.9%, *P* < 0.001). Patients with AKI more frequently received IMV (87.0% vs. 32.2%, *P* < 0.001) but less frequently received COT (2.2% vs. 33.1%, *P* < 0.001) and NIV (4.4% vs. 21.2%, *P* < 0.001). Furthermore, patients with AKI had markedly higher levels of serum creatinine (median: 75.5 vs. 67.1 μmol/L, *P* = 0.002), interleukin-6 (median: 17.9 vs. 12.3 pg/ml, *P* < 0.001) and serum ferritin (median: 2001.0 vs. 985.4 ng/ml, *P* < 0.001). Hypoxemia and hypercapnia were also more frequently identified in patients with AKI (both *P* < 0.001). In the multivariate regression model, greater age (OR 1.05, 95%CI: 1.01–1.09), sepsis (OR: 2.82, 95%CI: 1.14–6.98), use of nephrotoxic drug (OR: 2.67, 95%:1.09–6.55), IMV (OR: 9.72, 95%CI: 2.93–32.24) and elevated baseline serum creatinine levels (OR: 1.01, 95%CI: 1.00–1.02) were associated with AKI occurrence among patients with Covid-19. (Table 2).

**Table 2:** Risk factors associated with acute kidney injury in the multivariate regression analysis among patients with Covid-19

Variables	β	***p*** value	OR (95% CI)
**Age**	0.05	0.0064	1.05 (1.01–1.09)
**Sepsis**	1.04	0.0250	2.82 (1.14–6.98)
**Use of nephrotoxic drugs**	0.98	0.0316	2.67 (1.09–6.55)
**Respiratory support: invasive mechanical ventilation**	2.27	0.0002	9.72 (2.93–32.24)
**Baseline serum creatinine level**	0.01	0.0018	1.01 (1.00–1.02)

95%CI: 95% confident interval

### Outcomes of renal diseases and impact of AKI on clinic outcomes

**Fig. 1:**
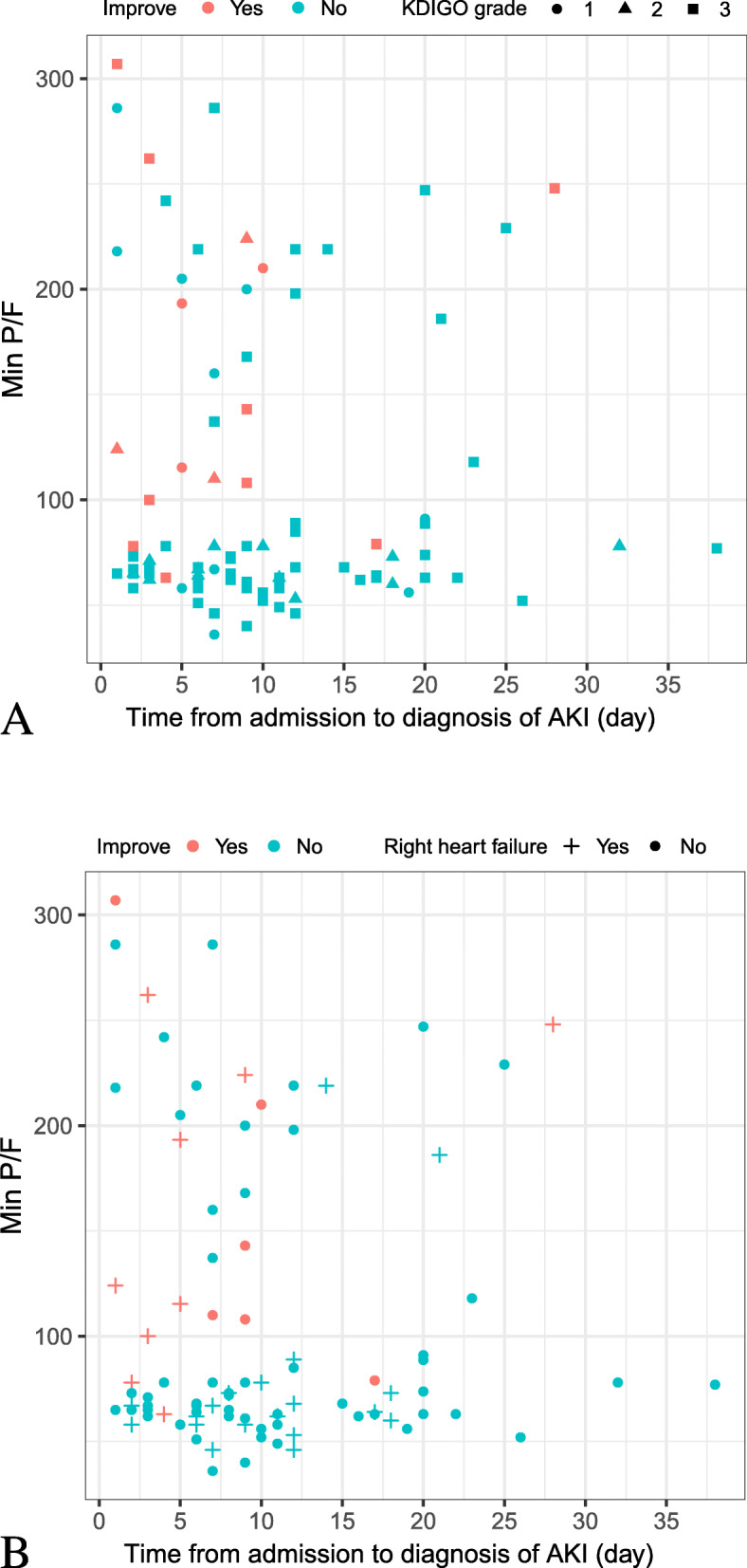
Association between the minimal PaO_2_/FiO_2_ ratio, time from admission to AKI diagnosis, right heart failure and the outcomes of renal disorders in patients with Covid-19 who had acute kidney injury during hospitalization. **a**. Association between the minimal PaO_2_/FiO_2_ ratio, time from admission to AKI diagnosis and the outcomes of renal disorder and the severity of acute kidney injury in patients with Covid-19 during hospitalization. **b**. Association between the minimal PaO_2_/FiO_2_ ratio, time from admission to AKI diagnosis and the outcomes of renal disorders and the presence of right heart failure in patients with Covid-19 who had acute kidney injury during hospitalization. KDIGO: Kidney Disease: Improving Global Outcomes. Min P/F: Minimal PaO_2_/FiO_2_ ratio

Of 92 patients with AKI, the renal function improved during hospitalization among 16 patients (17.4%) (one patient with renal recovery v.s.15 patients with renal non-recovery), who had a significantly shorter time from admission to AKI diagnosis (median: 5 vs. 9 days, *P* < 0.001), lower incidence of right heart failure (56.3% vs. 25.0%, *P* = 0.030) and higher PaO_2_/FiO_2_ ratio (median: 133.5 vs. 67.0, *P* < 0.001) than their counterparts (Table 3) (Fig. 1).

**Table 3:** Clinical characteristics of patients with AKI who had improved renal function and those without during hospitalization

Characteristic	Patients with AKI(***N*** = 92)	Improved(***n*** = 16)	Not improved(***n*** = 76)	***P*** value
**Age, median (IQR), y**	65 (59–73)	63 (49–66)	65 (61–74)	0.065
**Time from admission to diagnosis of AKI, (day)**	**9 (5–12)**	**5 (3–9)**	**9 (6–14)**	**< 0.001**
**Sex**				0.059
Female	30 (32.6)	2 (12.5)	28 (36.8)	–
Male	62 (67.4)	14 (87.5)	48 (63.2)	–
**KDIGO staging**				
1	13 (14.1)	3 (18.8)	10 (13.2)	0.850
2	15 (16.3)	3 (18.8)	12 (15.8)	> 0.999
3	64 (69.6)	10 (62.5)	54 (71.1)	0.706
**Comorbidities**				
Hypertension	45 (48.9)	8 (50.0)	37 (48.7)	0.924
Diabetes	23 (25.0)	6 (37.5)	17 (22.4)	0.341
Cardiovascular diseases	9 (9.8)	0 (0.0)	9 (11.8)	0.324
Malignancy	10 (10.9)	2 (12.50)	8 (10.5)	0.651
Cerebrovascular disease	12 (5.7)	12 (6.2)	0 (0.0)	> 0.999
Chronic Kidney Disease	6 (6.5)	3 (18.8)	3 (4.0)	0.063
Chronic Obstructive Pulmonary Disease	2 (2.2)	1 (6.3)	1 (1.3)	NA
Connective Tissue Disease	2 (2.2)	0 (0.0)	2 (2.6)	NA
**Complication**				
Sepsis	72 (78.3)	10 (62.5)	62 (81.6)	0.178
Right heart failure^a^	**28 (30.4)**	**9 (56.3)**	**19 (25.0)**	**0.030**
Disseminated Intravascular Coagulation	27 (29.4)	5 (31.3)	22 (29.0)	> 0.999
**Treatment**				
CRRT	52 (56.5)	9 (56.3)	43 (56.6)	0.981
Drug with Nephrotoxicity	38 (41.3)	7 (43.8)	31 (40.8)	0.827
Respiratory support: COT	2 (2.2)	1 (6.3)	1 (1.3)	NA
Respiratory support: HFN	6 (6.5)	0 (0.0)	6 (7.9)	NA
Respiratory support: NIV	4 (4.4)	1 (6.25)	3 (4.0)	NA
Respiratory support: IMV	80 (87.0)	14 (87.5)	66 (86.8)	> 0.999
**Laboratory Findings**^b^				
Baseline serum creatinine (μmol/L)	75.5 (59.8–102.8)	88.6 (54.4–229.8)	72.3 (61.8–90.8)	0.169
Maximum PaCO_2_ (mmHg)	69.5 (46.8–81.3)	65.5 (51.7–76.0)	72.0 (46.8–82.0)	0.881
Maximum PaCO_2_ > = 60 mmHg	55 (59.8)	9 (56.3)	46 (60.5)	0.751
Minimum PaO_2_/FiO_2_ ratio (mmHg)	**73.0 (62.8–138.6)**	**133.5 (106.0–230.0)**	**67.0 (62.0–88.8)**	**< 0.001**
Minimum PaO_2_/FiO_2_ ratio < 150 mmHg	70 (76.1)	9 (56.3)	61 (80.3)	0.085
Maximum IL-6 (pg/ml)	17.9 (12.6–33.5)	18.0 (14.0–97.3)	17.9 (12.1–31.5)	0.323
Maximum serum ferritin (ng/ml)	2001.0 (1329.6–2001.0)	2001.0 (1922.2–2001.0)	2001.00 (1296.5–2001.0)	0.889

Data in bold indicated the comparisons with statistical significance

^a^ Right heart failure: defined according to the clinical diagnosis and ultrasonographic manifestations. & Drugs with Nephrotoxicity: mainly included aminoglycosides, glycopeptides and colistin. ^b^, Data were collected before the development of AKI among patients who had developed AKI; The most abnormal data during hospitalization were presented among patients who did not develop AKI. PaCO_2_: arterial partial pressure of carbon dioxide; PaO_2_/FiO_2_ ratio: the ratio between the arterial partial pressure of oxygen and the inspiratory concentration of oxygen

**Fig. 2:**
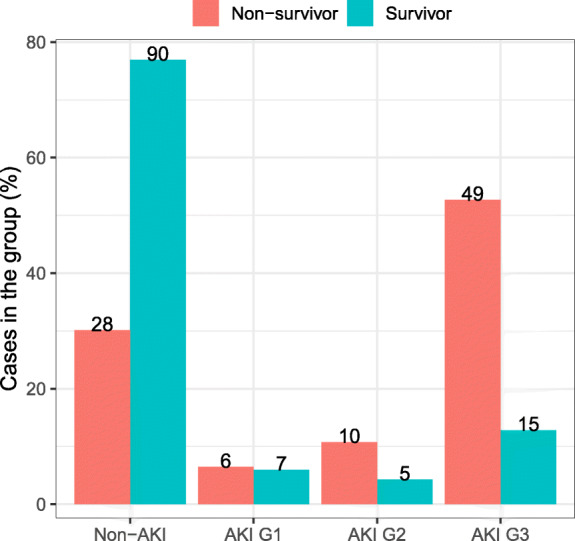
The proportion of patients with Covid-19 when stratified according to the survival status and the staging of the acute kidney injury. AKI: Acute kidney injury; G: stage

Of 210 patients, 93 deceased within 28 days of ICU admission (ICU 28-day mortality: 44.3%), and non-survivors were more likely to be critical cases compared with severe cases (98.9% vs. 1.1%, *P* < 0.001). Significantly more non-survivors had sepsis (77.4% vs. 34.2%, *P* < 0.001) acute kidney injury stage 3 (52.7% vs. 12.8%, *P* < 0.001) and disseminated intravascular coagulation (28.0% s. 5.1%, *P* < 0.001). CRRT was initiated significantly more frequently in non-survivors than in survivors (41.9% vs. 12.8%, *P* < 0.001). IMV was initiated more frequently whereas COT and HFNC were initiated less frequently in critical cases (all *P* < 0.01). Non-survivors had higher levels of interleukin-6 (median: 17.2 vs. 13.6 pg/ml, *P* = 0.031) and serum ferritin (median: 2001.0 vs. 1000.7 ng/ml, *P* < 0.001) compared with survivors. Hypoxemia and hypercapnia were also more common in non-survivors (both *P* < 0.001). However, neither the use of nephrotoxic drugs nor the serum creatinine levels differed between survivors and non-survivors (Table 4). After adjusting for the potential confounding factors, having KDIGO stage 3 (OR: 5.33, 95%CI: 1.15–24.65), critical illness (OR: 69.16, 95%CI: 5.86–815.79), greater age (OR: 1.06, 95%CI: 1.02–1.11) and the lowest PaO_2_/FiO_2_ < 150 mmHg (OR: 15.21, 95%CI: 4.72–49.07) independently predicted the death within 28 days of ICU admission (Table 5, Fig. 2).

**Table 4:** Clinical characteristics of patients with AKI who had survived and those not

Characteristic	Total(***N*** = 210)	Non-survivor(***n*** = 93)	Survivor(***n*** = 117)	***P*** value
**Age, median (IQR), yrs**	**64 (56–71)**	**66 (61–73)**	**62 (53–69)**	**0.002**
**Sex**				0.997
Female	79 (37.6)	35 (37.6)	44 (37.6)	–
Male	131 (62.4)	58 (62.4)	73 (62.4)	–
**Severity of Covid-19**				**< 0.001**
Severe	**60 (28.6)**	**1 (1.1)**	**59 (50.4)**	–
Critical	**150 (71.4)**	**92 (98.9)**	**58 (49.6)**	–
**Comorbidities**				
Hypertension	98 (46.7)	43 (46.2)	55 (47.0)	0.911
Diabetes	44 (21.0)	21 (22.6)	23 (19.7)	0.605
Cardiovascular diseases	23 (11.0)	10 (10.8)	13 (11.1)	0.934
Malignancy	14 (6.7)	9 (9.7)	5 (4.3)	0.119
Cerebrovascular disease	12 (5.7)	7 (7.5)	5 (4.3)	0.313
Chronic Kidney Disease	10 (4.8)	3 (3.2)	7 (6.0)	0.545
Chronic Obstructive Pulmonary Disease	5 (2.4)	2 (2.2)	3 (2.6)	NA
Connective Tissue Disease	2 (1.0)	1 (1.1)	1 (0.9)	NA
**Complication**				
Sepsis	**112 (53.3)**	**72 (77.4)**	**40 (34.2)**	**< 0.001**
Acute kidney injury	**92 (43.8)**	**65 (69.9)**	**27 (23.1)**	**< 0.001**
AKI KDIGO stage 1	13 (6.2)	6 (6.5)	7 (6.0)	0.889
AKI KDIGO stage 2	15 (7.1)	10 (10.8)	5 (4.3)	0.070
AKI KDIGO stage 3	**64 (30.5)**	**49 (52.7)**	**15 (12.8)**	**< 0.001**
Right heart failure^a^	44 (21.0)	25 (26.9)	19 (16.2)	0.060
Disseminated Intravascular Coagulation	**32 (15.2)**	**26 (28.0)**	**6 (5.1)**	**< 0.001**
**Treatment**				
Continuous renal replacement therapy	**54 (25.7)**	**39 (41.9)**	**15 (12.82)**	**< 0.001**
Drug with Nephrotoxicity	52 (24.8)	28 (30.1)	24 (20.51)	0.110
Respiratory support: COT	41 (19.5)	0 (0.00)	41 (35.04)	0.287
Respiratory support: HFN	**22 (10.5)**	**3 (3.23)**	**19 (16.24)**	**0.002**
Respiratory support: NIV	29 (13.8)	10 (10.75)	19 (16.24)	0.252
Respiratory support: IMV	**118 (56.2)**	**80 (86.0)**	**38 (32.5)**	**< 0.001**
**Laboratory Findings**				
Baseline Scr(umol/L)	69.7 (55.9–85.9)	71.1 (58.4–90.8)	69.0 (54.4–81.3)	0.165
Maximum PaCO_2_(mmHg)	**52.0 (39.0–73.8)**	**71.0 (54.0–79.0)**	**43.0 (38.0–54.0)**	**< 0.001**
Maximum PaCO_2_ > = 60 mmHg	**82 (39.1)**	**61 (65.6)**	**21 (18.0)**	**< 0.001**
Minimum P/F(mmHg)	**109.0 (65.0–207.7)**	**65.0 (58.0–75.9)**	**190.0 (130.0–220.0)**	**< 0.001**
Minimum P/F < 150 mmHg	**116 (55.2)**	**82 (88.2)**	**34 (29.1)**	**< 0.001**
Maximum IL-6 (pg/ml)	**14.3 (9.9–24.9)**	**17.2 (11.7–31.2)**	**13.6 (9.3–22.2)**	**0.031**
Maximum serum ferritin (ng/ml)	**1607.8 (786.2–2001.0)**	**2001.0 (1130.0–2001.0)**	**1000.7 (590.2–2001.0)**	**< 0.001**

Right heart failure: defined according to the clinical diagnosis and ultrasonographic manifestations. & Drugs with Nephrotoxicity: mainly included aminoglycosides, glycopeptides and colistin. ^a^, Data were collected before the development of AKI among patients who had developed AKI; The most abnormal data during hospitalization were presented among patients who did not develop AKI. PaCO_2_: arterial partial pressure of carbon dioxide; PaO_2_/FiO_2_ ratio: the ratio between the arterial partial pressure of oxygen and the inspiratory concentration of oxygen

Data in bold indicated the comparisons with statistical significance

Non-survivor: Deceased within 28 days of ICU admission

**Table 5:** Risk factors associated with deceased within 28 days of ICU admission in the multivariate regression analysis among patients with Covid-19

Variables	β	***p*** value	OR (95% CI)
**Age**	0.06	0.0035	1.06 (1.02–1.11)
**Severity of Covid-19: critical**	4.24	0.0008	69.16 (5.86–815.79)
**AKI stage 3**	1.67	0.0321	5.33 (1.15–24.65)
**Min P/F < 150 mmHg**	2.72	0.0000	15.21 (4.72–49.07)

*95%CI* 95% confident interval

*Min P/F* Minimal PaO_2_/FiO_2_ ratio

## Discussion

In this study, we sought to investigate the association between AKI and the clinical outcomes in severe and critical patients with Covid-19. We have found that, among patients with Covid-19, the incidence of any AKI stages, AKI stage 1, 2 and 3 was 43.8, 14.1, 16.3 and 69.6%, respectively. Age, the presence of sepsis, use of nephrotoxic drug, IMV and baseline serum creatinine levels were associated with the development of AKI. 58.7% of patients received CRRT, and only 17.4% of patients with AKI achieved recovery of the renal function. Furthermore, the time from admission to AKI diagnosis, right heart failure and PaO_2_/FiO_2_ ratio were independently associated with the renal recovery. All patients with AKI who required continuous hemodialysis were treated with CRRT. Furthermore, greater age, severe hypoxemia, having severe AKI and the development of critical Covid-19 independently predicted the risk of death within 28 days of ICU admission.

AKI has been one of the most frequent complications among patients admitted to the ICU. A 2004–2012 systematic review that included large-scale cohort studies has estimated the global incidence of AKI to be 21.6% in adults (95%CI, 19.3–24.1), and 31.7% (95%CI, 28.6–35.0) in critical care settings, according to the KDIGO guideline [14]. However, a recent study which enrolled 634 patients with ARDS demonstrated that an incidence rate of 68.3% for AKI after the onset of ARDS [15]. In another observational study on the critically ill patients with 2009 influenza A (H1N1) viral pneumonia, the incidence of AKI reached to 51% [16]. Our finding of the higher incidence of AKI (43.8%) in patients with Covid-19 therefore might have been attributable to the high incidence of ARDS during hospitalization.

Previous studies had demonstrated that greater age, sepsis and baseline serum creatinine levels was associated with the development of AKI in adult patients [10, 15, 17–21], which was consistent with our results. Recently, the association between IMV and AKI in ARDS patients have become widely acceptable. Several studies had postulated that IMV might have played a key role in the development of AKI due to the mechanisms of lung-kidney cross-talk [22, 23], and lung protective ventilation strategy including low tidal volume ventilation, limited plateau pressure, and suitable positive end-expiratory pressure level was postulated to decrease the risk of AKI [24]. However, hypercapnia was very common in our patients, especially the patients with AKI. It was challenging to correct for the hypercapnia even the patients had been intubated and receiving lung protective ventilation strategy. Physicians may adjust the tidal volume and plateau pressure in a very constrained environment, especially at the early stage of epidemic. This might explain why IMV was the strongest risk factors associated with AKI.

In our study, only 16 AKI patients (17.4%) achieved recovery of renal function during hospitalization, which was significantly lower than that reported previously (39.3%) [25]. Our analysis revealed that the delayed onset of AKI, the presence of right heart failure and lower PaO_2_/FiO_2_ ratio were associated with an increased likelihood of having non-recovery of renal function. Patients with a delayed onset AKI might have a prolonged course of hospital management, including hemodynamic deterioration, use of nephrotoxic drugs and the adverse impacts associated with lung-renal cross-talk during the course of ARDS development [18, 19]. Severe hypoxemia had been considered as a cause of chronic kidney disease and AKI [26–28].The severity and duration of AKI might depend on the severity and duration of hypoxemia. In our study, the overall incidence of right heart failure was similar with that reported in a previous study [29]. Persistent right heart failure would lead to elevated central venous pressure and peripheral edema, which were risk factors of AKI in critically illness [30].

Several previous studies had demonstrated that AKI, especially severe AKI was associated with poorer clinical outcomes [15, 18, 31]. The AKI-EPI study showed that patients with stage 1 AKI did not have a higher risk of mortality compared to patients without AKI [32]. However, in our study, only AKI KDIGO stage 3 was associated with ICU 28-day mortality, patients with KDIGO stage 1 and 2 AKI did not have a higher mortality compared to patients without AKI.

To our knowledge, this is the first and largest study which investigates the incidence, risk factors of AKI, and the association between AKI and clinical outcomes in severe and critical patients with Covid-19. However, we acknowledge a number of limitations of the current study, which are in part due to the nature of retrospective study. We did not collect the ventilator parameters which might play a key role in lung-kidney crosstalk. We also did not assess the fluid balance per day due to the very constrained environment. Therefore, findings of our study needed further validation.

## Conclusion

In conclusion, among patients with Covid-19, the incidence of AKI was high. Age, sepsis, nephrotoxic drug, invasive mechanical ventilation and baseline serum creatinine were strongly associated with the development of AKI. Time from admission to AKI diagnosis, right heart failure and PaO_2_/FiO_2_ ratio were independently associated with the potential of renal recovery. Finally, AKI KIDGO stage 3 independently predicted the risk of death within 28 days of ICU admission. Our findings of the risk factors of the development of AKI and factors associated with renal function recovery may inform clinical management of patients with critical illness of Covid-19.

## Supplementary information

**Additional file 1: Table E1.** Clinical characteristics of patients with Covid-19 when stratified by the disease severity. **Table E2.** Patient’s characteristics between the two hospitals.

## Data Availability

Data sharing will be considered only on a collaborative basis with the principal investigators, after evaluation of the proposed study protocol and statistical analysis plan. Data can be obtained by contacting the first author(LS, sonysang999@vip.163.com) or correspondent author(YML, dryiminli@vip.163.com).

## Abbreviations

Covid-19: Corona virus disease 2019
SARS-CoV-2: Severe acute respiratory syndrome coronavirus 2
ARDS: Acute respiratory distress syndrome
AKI: Acute kidney injury
ICU: Intensive care unit
MV: Mechanical ventilation
CRRT: Continuous renal replacement therapy
P/F: PaO_2_/FiO_2_
KIDGO: Kidney disease: improving global outcomes
ADQI: Acute disease quality initiative
IQRs: Interquartile ranges
OR: Odds ratio
COT: Conventional oxygen therapy
HFNC: High-flow nasal cannula
IMV: Invasive mechanical ventilation
NIV: Non-invasive mechanical ventilation
ETA: Endotracheal aspirate
