# Supplementary material for: The incidence, risk factors and prognosis of acute kidney injury in severe and critically ill patients with COVID-19 in mainland China: a retrospective study
Source: BMC Pulm Med. 2020 Nov 9;20:290. doi: 10.1186/s12890-020-01305-5 (PMC7649893; doi:10.1186/s12890-020-01305-5)
Supplement: Supplementary file 1 — Additional file 1: Table E1. Clinical characteristics of patients with Covid-19 when stratified by the disease severity. Table E2. Patient’s characteristics between the two hospitals. [file 12890_2020_1305_MOESM1_ESM.doc]

**Online supplement（Results）**

**The incidence, risk factors and prognosis of acute kidney injury in severe and critically ill patients with COVID-19 in mainland China: a retrospective study**

**Results**

***Patient’s characteristics between the two hospitals***

Of the 210 patients, 194 (92.4%) were recruited from Wuhan JinYinTan Hospital, whereas the remaining 16 (7.6%) were recruited from The First Affiliated Hospital of Guangzhou Medical University. Overall, there were no significant differences in terms of the patient’s demographic characteristics, disease severity, comorbidities and treatment between the two hospitals. However, patients from Wuhan JinYinTan Hospital were more likely to have right heart failure, hypercapnia, hypoxemia and lower levels of serum interleukin-6 than the patients from The First Affiliated Hospital of Guangzhou Medical University (**Table E2**).

**Table E1. Clinical characteristics of patients with Covid-19 when stratified by the disease severity**

| **Characteristic** | **Total (N = 210)** | **Severe cases (N = 60)** | **Critical cases (N = 150)** | ***P* value** |
| --- | --- | --- | --- | --- |
| **Age, median (IQR), yrs** | 64 (56-71) | 63 (53-70) | 64 (56-71) | 0.631 |
| **Sex – No., %** |  |  |  | 0.444 |
| Female | 79 (37.6) | 25 (41.7) | 54 (36.0) | - |
| Male | 131 (62.4) | 35 (58.3) | 96 (64.0) | - |
| **Comorbidities** **– No., %** |  |  |  |  |
| Hypertension | 98 (46.7) | 29 (48.3) | 69 (46.0) | 0.760 |
| Diabetes | **44 (21.0)** | **7 (11.7)** | **37 (24.7)** | **0.037** |
| Cardiovascular diseases | 23 (11.0) | 9 (15.0) | 14 (9.3) | 0.235 |
| Malignancy | 14 (6.7) | 1 (1.7) | 13 (8.7) | 0.126 |
| Cerebrovascular disease | 12 (5.7) | 3 (5.0) | 9 (6.0) | >0.999 |
| Chronic Kidney Disease* | 10 (4.8) | 3 (5.0) | 7 (4.7) | >0.999 |
| Chronic Obstructive Pulmonary Disease! | 5 (2.4) | 1 (1.7) | 4 (2.7) | >0.999 |
| Connective Tissue Disease# | 2 (1.0) | 0 (0.0) | 2 (1.3) | >0.999 |
| **Complications** |  |  |  |  |
| Sepsis | **112 (53.3)** | **3 (5.0)** | **109 (72.7)** | **<0.001** |
| Right heart failure$ | **44 (21.0)** | **2 (3.3)** | **42 (28.0)** | **0.0001** |
| Disseminated Intravascular Coagulation% | **32 (15.2)** | **0 (0.0)** | **32 (21.3)** | **0.0001** |
| **Treatment** |  |  |  |  |
| Drugs with nephrotoxicity& | **52 (24.7)** | **2 (3.3)** | **50 (33.3)** | **<0.001** |
| Respiratory support: COT | **41 (19.5)** | **39 (65.0)** | **2 (1.33)** | **<0.001** |
| Respiratory support: HFNC | **22 (10.5)** | **12 (20.0)** | **10 (6.7)** | **0.004** |
| Respiratory support: NIV | 29 (13.8) | 7 (11.7) | 22 (14.7) | 0.569 |
| Respiratory support: IMV | **118 (56.2)** | **2 (3.3)** | **116 (77.3)** | **<0.001** |
| **Laboratory Findings^** |  |  |  |  |
| Baseline serum creatinine (μmol/L) | 69.7 (55.9-85.9) | 66.1 (56.5-75.7) | 72.3 (55.0-90.5) | 0.079 |
| Maximum PaCO_2_ (mmHg) | **52.0 (39.0-73.8)** | **39.5 (37.0-45.0)** | **62.0 (45.0-78.0)** | **<0.001** |
| Maximum PaCO_2_ >= 60 mmHg | **82 (39.05)** | **1 (1.67)** | **81 (54.00)** | **<0.001** |
| Minimum PaO_2_/FiO_2_ ratio (mmHg) | **109.0 (65.0-207.7)** | **206.5 (166.0-220.0)** | **73.9 (62.3-164.5)** | **<0.001** |
| Minimum PaO_2_/FiO_2_ ratio <150 mmHg | **116 (55.24)** | **8 (13.33)** | **108 (72.00)** | **<0.001** |
| Maximum serum IL-6 (pg/ml) | **14.3 (9.9-24.9)** | **9.9 (8.3-14.7)** | **17.2 (11.7-32.6)** | **<0.001** |
| Maximum serum ferritin (ng/ml) | **1607.8 (786.2-2001.0)** | **785.4 (454.9-1326.2)** | **2001.0 (1124.9-2001.0)** | **<0.001** |

$ Right heart failure: defined according to the clinical diagnosis and ultrasonographic manifestations. & Drugs with Nephrotoxicity: mainly included aminoglycosides, glycopeptides and colistin. ^, Data were collected before the development of AKI among patients who had developed AKI; The most abnormal data during hospitalization were presented among patients who did not develop AKI. PaCO_2_: arterial partial pressure of carbon dioxide; PaO_2_/FiO_2_ ratio: the ratio between the arterial partial pressure of oxygen and the inspiratory concentration of oxygen

Data in bold indicated the comparisons with statistical significance.

**Table E2. Patient’s characteristics between the two hospitals**

| **Characteristic** | **Total (N = 210)** | **JYTH (n = 194)** | **GZMUH (n = 16)** | ***P* value** |
| --- | --- | --- | --- | --- |
| **Age, median (IQR), y** | 64 (56-71) | 64 (56-71) | 58 (49-67) | 0.165 |
| **Sex** |  |  |  | 0.278 |
| Female | 79 (37.6) | 75 (38.7) | 4 (25.0) | - |
| Male | 131 (62.4) | 119 (61.3) | 12 (75.0) | - |
| **Severity of Covid-19** |  |  |  | 0.077 |
| Severe | 60 (28.6) | 59 (30.4) | 1 (6.3) | - |
| Critical | 150 (71.4) | 135 (69.6) | 15 (93.8) | - |
| **Comorbidities** |  |  |  |  |
| Hypertension | 98 (46.7) | 91 (46.9) | 7 (43.8) | 0.808 |
| Diabetes | 44 (21.0) | 38 (19.6) | 6 (37.5) | 0.170 |
| Cardiovascular diseases | 23 (11.0) | 23 (11.9) | 0 (0.0) | 0.297 |
| Malignancy | 14 (6.7) | 12 (6.2) | 2 (12.5) | 0.651 |
| Cerebrovascular disease | 12 (5.7) | 12 (6.2) | 0 (0.0) | NA |
| Chronic Kidney Disease* | 10 (4.8) | 10 (5.2) | 0 (0.0) | NA |
| Chronic Obstructive Pulmonary Disease! | 5 (2.4) | 4 (2.1) | 1 (6.3) | NA |
| Connective Tissue Disease# | 2 (1.0) | 2 (1.0) | 0 (0.0) | NA |
| **Complications** |  |  |  |  |
| Sepsis | 112 (53.3) | 103 (53.09) | 9 (56.3) | 0.808 |
| Right heart failure$ | **44 (21.0)** | **36 (18.6)** | **8 (50.0)** | **0.008** |
| Disseminated Intravascular Coagulation% | 32 (15.2) | 32 (16.5) | 0 (0.0) | 0.161 |
| **Treatment** |  |  |  |  |
| Drugs with nephrotoxicity& | 52 (24.8) | 50 (25.8) | 2 (12.5) | 0.378 |
| Respiratory support: COT | 41 (19.5) | 40 (20.6) | 1 (6.3) | 0.287 |
| Respiratory support: HFNC | 22 (10.5) | 22 (11.3) | 0 (0.0) | 0.318 |
| Respiratory support: NIV | 29 (13.8) | 26 (13.4) | 3 (18.8) | 0.827 |
| Respiratory support: IMV | 118 (56.2) | 106 (54.6) | 12 (75.0) | 0.115 |
| **Laboratory Findings^** |  |  |  |  |
| Baseline serum creatinine (μmol/L) | 69.7 (55.9-85.9) | 69.0 (55.3-85.6) | 75.6 (61.7-92.1) | 0.287 |
| Maximum PaCO_2_ (mmHg) | **52.0 (39.0-73.8)** | **54.0 (39.0-75.0)** | **41.5 (38.2-49.4)** | **0.025** |
| Maximum PaCO_2_ >= 60 mmHg | **82 (39.1)** | **81 (41.8)** | **1 (6.3)** | **0.005** |
| Minimum PaO_2_/FiO_2_ ratio (mmHg) | **109.0 (65.0-207.7)** | **90.5 (64.3-205.0)** | **181.6 (126.3-211.5)** | **0.016** |
| Minimum PaO_2_/FiO_2_ ratio <150 mmHg | 116 (55.2) | 109 (56.2) | 7 (43.8) | 0.336 |
| Maximum serum IL-6 (pg/ml) | **14.3 (9.9-24.9)** | **13.8 (9.7-21.6)** | **104.5 (64.0-364.5)** | **<0.001** |
| Maximum serum ferritin (ng/ml) ¥ | 1607.8 (786.2-2001.0) | 1607.8 (786.2-2001.0) | NA | NA |

Right heart failure: defined according to the clinical diagnosis and ultrasonographic manifestations. & Drugs with Nephrotoxicity: mainly included aminoglycosides, glycopeptides and colistin. ^, Data were collected before the development of AKI among patients who had developed AKI; The most abnormal data during hospitalization were presented among patients who did not develop AKI. PaCO_2_: arterial partial pressure of carbon dioxide; PaO_2_/FiO_2_ ratio: the ratio between the arterial partial pressure of oxygen and the inspiratory concentration of oxygen

Data in bold indicated the comparisons with statistical significance.

¥ Not performed in GZMUH

JYTH: Wuhan JinYinTan Hospital; GZMUH: The First Affiliated Hospital of Guangzhou Medical University; NA: Not applicable
